# Therapeutic Effects of Argon Inhalation on Lung Ischemia–Reperfusion Injury in CLAWN Miniature Swine

**DOI:** 10.3390/jcm14248821

**Published:** 2025-12-12

**Authors:** Takehiro Iwanaga, Masayoshi Okumi, Yuichi Ariyoshi, Kazuhiro Takeuchi, Akira Kondo, Mitsuhiro Sekijima, Yurika Ichinari, Akira Shimizu, Hisashi Sahara

**Affiliations:** 1 Division of Experimental Large Animal Research, Life Science and Laboratory Animal Research Unit, Center for Advanced Science Research and Promotion, Kagoshima University, Kagoshima 890-8520, Japan; tiwanaga.arc@tmd.ac.jp (T.I.);; 2 Animal Research Facilities, Bioscience Center, Research Infrastructure Management Center, Institute of Science Tokyo, Tokyo 113-8510, Japan; 3 Department of Urology, Kyoto Prefectural University of Medicine, Kyoto 602-8566, Japan; okumi@koto.kpu-m.ac.jp; 4 Department of Analytic Human Pathology, Nippon Medical School, Tokyo 113-8602, Japan

**Keywords:** ischemia–reperfusion injury, argon, miniature swine, large animal model, lung transplantation, warm ischemia, antioxidant, anti-apoptosis, noble gas

## Abstract

**Background**: Noble gases, such as argon, have been observed to exhibit cytoprotective effects. The non-anesthetic properties, abundance, and cost-effectiveness of argon suggest its clinical potential. While its efficacy in mitigating ischemia–reperfusion injury has been demonstrated in cellular and small animal models, data on its effects in large animals remain limited. This study evaluated the effects of argon inhalation on pulmonary ischemia–reperfusion injury in miniature swine with potential applications in transplantation. **Methods**: The left bronchial and pulmonary artery and veins were clamped for 90 min, and then the clamps were released to induce lung ischemia–reperfusion injury in 10 CLAWN miniature swine. The argon group (n = 5) inhaled a mixture of 30% oxygen and 70% argon for 360 min, whereas the control group (n = 5) inhaled a mixture of 30% oxygen and 70% nitrogen for an equivalent duration. Lung function was evaluated using chest X-ray, lung biopsies, and blood gas analysis. **Results**: The PaO_2_/FiO_2_ ratio significantly decreased in the control group 2 h post-reperfusion (568 ± 12 to 272 ± 39 mmHg), but was better preserved in the argon group (562 ± 17 to 430 ± 48 mmHg). Blood gas from the left pulmonary vein showed a superior PvO_2_/FiO_2_ ratio in the argon group (331 ± 40 vs. 186 ± 17 mmHg at 2 h; 519 ± 19 vs. 292 ± 33 mmHg at 2 days). Chest X-ray revealed reduced infiltration in the left lung. The lung biopsy histological scores improved in the argon group at 2 h and 2 days. Serum superoxide dismutase analysis and tissue TUNEL assays suggested that antioxidant and anti-apoptotic mechanisms, respectively, were involved. **Conclusions**: Perioperative argon inhalation attenuates ischemia–reperfusion injury in swine lungs, likely via anti-apoptotic and antioxidant effects.

## 1. Introduction

Organ transplantation is widely recognized as an effective treatment for end-stage organ failure. However, the persistent shortage of donor organs remains a major obstacle [1]. According to the Global Observatory on Donation and Transplantation, 172,409 solid organ transplants were performed worldwide in 2023, supported by 45,861 deceased organ donors, including those who donated after brain death and those who donated after circulatory death [2]. However, the demand for transplantation continues to exceed the available supply [3].

To expand the donor pool, organs from marginal donors, such as elderly individuals or those with hypertension or diabetes, are increasingly being utilized [4]. DCD donors are an important source of organs, in addition to conventional brain-dead and living donors. This approach has emerged as a viable strategy to expand the donor pool [5]. However, in DCD donors, the inevitable period of warm ischemia due to circulatory arrest markedly increases the risk of ischemia–reperfusion injury (IRI) [6,7]. IRI involves inflammation, oxidative stress, and tissue damage triggered by the temporary cessation and subsequent restoration of blood flow [8]. It contributes to primary graft dysfunction (PGD) and promotes immune activation, leading to graft rejection and chronic lung allograft dysfunction (CLAD) [9]. Despite extensive research, clinically applicable pharmacological strategies for mitigating IRI remain limited.

Given this unmet need, attention has turned to cytoprotective agents, including noble gases. Helium, argon (Ar), and xenon are chemically inert elements, known as noble gases, found in trace amounts in the atmosphere [10] and have been explored in industrial and medical contexts [11,12]. Among these, Ar is relatively abundant, non-anesthetic, and cost-effective, making it particularly attractive for potential therapeutic use. In contrast, helium is scarce and xenon is both expensive and anesthetic [13].

Experimental studies in small animal models have shown that the biological effects of Ar vary across organs. In the heart, Ar exposure reduces infarct size and improves post-ischemic recovery [14,15]. In the kidney, the use of Ar-saturated preservation solutions ameliorated IRI in a transplantation model [16]. In contrast, hepatic studies have yielded less favorable results; inhaled Ar did not protect against liver injury or enhance regeneration in partial hepatectomy or warm IRI models [17,18]. Large animal studies similarly demonstrate organ-dependent variability in the protective effects of Ar. Reported benefits include reduced neuronal death in porcine cerebral ischemia [19] and improved renal function in porcine kidney perfusion models [20], whereas no protective effect was observed in a porcine ex vivo lung perfusion model [21]. Taken together, these findings indicate that the organ-specific responses observed in small animal studies extend to large animal models, and the overall efficacy of Ar across organ systems remains uncertain. Notably, evidence supporting protective effects against pulmonary IRI in clinically relevant large animal settings is still very limited.

Therefore, this study aimed to evaluate the efficacy and safety of perioperative Ar inhalation in a warm lung IRI model using CLAWN miniature swine. We examined pulmonary oxygenation, radiographic and histological injury, apoptosis, and oxidative stress markers to determine whether Ar inhalation can mitigate acute lung injury following IRI.

## 2. Materials and Methods

### 2.1. Animals

Ten CLAWN miniature swine (5–6 months old, 11.9–16.9 kg) were obtained from the Kagoshima Miniature Swine Research Center (Kagoshima, Japan). All procedures complied with the 3Rs principles (Replacement, Reduction, and Refinement), emphasizing animal welfare and appropriate housing. To minimize the number of animals used, the study was carefully designed and approved by the Kagoshima University Animal Experiment Committee (Protocol no. MD20016). All handling and procedures followed the Kagoshima University Guidelines for Animal Experimentation, the internationally accepted standards (NIH Publication No. 86-23, revised 1985), and the ARRIVE guidelines (2020 revision).

### 2.2. Experimental Groups and Gas Inhalation Protocol

Animals were divided into two groups: an Ar inhalation group (Ar group, n = 5) and a control group (n = 5). In the Ar group, a gas mixture containing 70% Ar and 30% O_2_ was used, whereas the control group received a gas mixture of 70% nitrogen and 30% O_2_. The concentration of 70% Ar was selected as the highest dose that could be safely administered while maintaining an FiO_2_ value of 0.30. Gas inhalation was maintained for 360 min to cover the entire peri-reperfusion period, starting from the beginning of the operation and continuing until 2 h post-reperfusion in both groups, based on our previous porcine lung IRI model [22].

### 2.3. Surgical Procedure

Anesthesia was induced by intramuscular injection of ketamine, midazolam, and medetomidine, followed by tracheal intubation and mechanical ventilation (100% O_2_, 12–15 mL/kg, 12–15 breaths/min). Anesthesia was maintained with 1–3% isoflurane. Catheters were inserted into the internal carotid artery (for blood pressure monitoring and gas analysis) and the external jugular vein (for sampling). The ventilator gas mixture was switched to the designated gas mixtures for each group.

**Figure 1:**
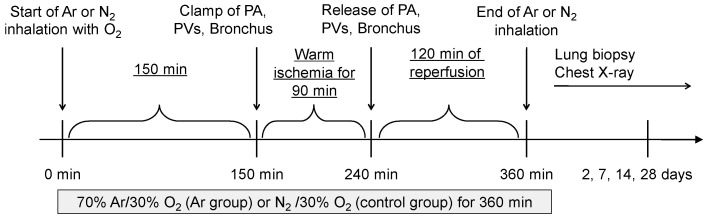
Schematic overview of the experimental protocol. Argon (70% Ar/30% O_2_) or control gas (70% N_2_/30% O_2_) inhalation was initiated at the beginning of the operation and maintained for a total of 360 min. At 150 min, the left pulmonary artery (PA), pulmonary veins (PVs), and main bronchus were clamped for 90 min to induce warm ischemia. Reperfusion was started at 240 min by releasing the clamps, followed by a 2 h reperfusion period. Gas inhalation was terminated at 360 min. Lung biopsy and chest radiography were performed 2, 7, 14, and 28 days post-reperfusion.

The lung IRI model was established as previously described [22]. Briefly, left fifth intercostal thoracotomy was performed to expose the pulmonary hilum. Then, 150 min after starting gas inhalation, the left pulmonary artery, veins, and main bronchus were clamped for 90 min and released to initiate reperfusion and establish a warm IRI model (Figure 1).

### 2.4. Blood Gas Measurements

To evaluate pulmonary function, arterial blood samples were collected from the carotid artery before surgery and 2 h post-reperfusion. Pulmonary venous blood was sampled from the left pulmonary vein 2 h and 2 days post-reperfusion. Blood gas analysis was performed using a blood gas analyzer (IL Japan, Tokyo, Japan).

The left pulmonary vein was selected because it drains blood directly from the reperfused left lung, providing an accurate reflection of the local oxygen exchange without dilution by the contralateral lung. Therefore, the pulmonary venous oxygenation index (PvO_2_/FiO_2_ ratio, abbreviated as Pv/F ratio) was calculated to assess regional lung oxygenation.

### 2.5. Chest X-Ray

Chest X-rays were obtained under general anesthesia on days 2, 7, 14, and 28 post-reperfusion to monitor lung injury.

### 2.6. Histological Evaluation

Lung tissue samples were collected at 2 h and on days 2, 7, 14, and 28 post-reperfusion, fixed in 10% neutral buffered formalin, and embedded in paraffin. The sections were stained with hematoxylin and eosin (H&E) and Elastica Masson–Goldner (EMG) for histopathological assessments. Histological injury was assessed using the semiquantitative scoring system previously reported [22], which was adapted from the criteria described by Müller et al. [23]. Briefly, cell infiltration, intra-alveolar edema, fibrin exudation, and hemorrhage were each graded from 0 (normal) to 3 (severe). Apoptotic cells were detected using the Terminal deoxynucleotidyl transferase-mediated UTP-biotin nick end labeling (TUNEL) assay. All histological assessment was conducted by a renal pathologist who was blinded to group allocation (A.S.).

### 2.7. Renal and Hepatic Function

To assess organ function, the serum creatinine (Cre) and alanine aminotransferase (ALT) levels were measured at baseline (pre-ischemia) and on days 2, 7, 14, and 28 post-reperfusion.

### 2.8. Inflammatory and Oxidative Markers

To evaluate systemic inflammation and oxidative stress, serum levels of interleukin-6 (IL-6) and superoxide dismutase (SOD) were measured. Blood was collected from the jugular vein before ischemia and 1, 2, and 6 h post-reperfusion for analysis. Measurements were conducted using ELISA kits (IL-6: R&D Systems, Minneapolis, MN, USA; SOD: Cayman Chemical, Ann Arbor, MI, USA). For quantitative assessment of apoptosis, TUNEL-positive nuclei in lung sections obtained 2 days post-reperfusion were counted in randomly selected high-power fields (400×). The mean number of TUNEL-positive cells per field was calculated for statistical comparison between groups.

### 2.9. Gene Expression Analysis

To evaluate the gene expression levels of Caspase-3 (apoptosis-related) and IL-6 (inflammatory cytokine), lung biopsy samples were collected 2 h and 2 days post-reperfusion. Total RNA was extracted using an RNeasy Midi Kit (QIAGEN, Hilden, Germany) according to the manufacturer’s protocol. Complementary DNA (cDNA) was synthesized using a PrimeScript RT reagent kit with DNA Eraser (Takara Bio, Kusatsu, Japan). Quantitative PCR was conducted using SYBR Premix Ex Taq (Takara Bio), and amplification was performed with a Thermal Cycler Dice Real-Time System II (Takara Bio). Ribosomal protein L4 (RPL4) was used as the reference gene for relative quantification and analyzed using Multiplate RQ software Ver 6.0.1 (Takara Bio). The relative expression levels were normalized to RPL4 and expressed as fold-changes relative to pre-ischemia values. The primer and probe sequences are as follows: Caspase-3 sense primer, 5′-AAGTTTCTTCAGAGGGGACTGC-3′; antisense primer, 5′-ACTGCTACCTTTCGGTTAACCC-3′; IL-6 sense primer, 5′-AAGCACTGATCCAGACCCTGAG-3′; antisense primer, 5′-TCAGGTGCCCCAGCTACATTAT-3′; RPL4 sense primer, 5′-TTTGTGGTAGGCTATGCCCTTG-3′; and antisense primer, 5′-CAATGGGACTCCAGATGTTTCC-3′.

### 2.10. Statistical Analysis

Data are presented as the mean ± standard error of the mean (SEM). Comparisons between the two groups at each time point were performed using an unpaired Student’s *t*-test. For within-group comparisons between baseline and post-reperfusion values, a paired *t*-test was used, and statistical analyses were performed using GraphPad Prism 6 (GraphPad Software, La Jolla, CA, USA). A *p*-value < 0.05 was considered statistically significant.

## 3. Results

### 3.1. Argon Inhalation Preserves Systemic and Pulmonary Oxygenation After Lung IRI

**Figure 2:**
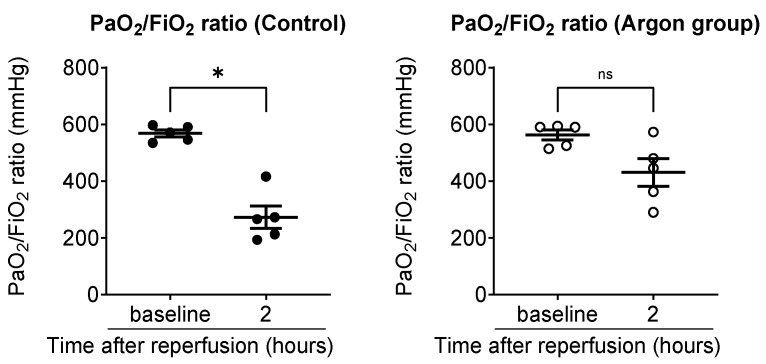
Comparison of arterial oxygenation between the control and argon (Ar) groups (black dots: control; white circles: Ar group). Arterial blood samples were collected from the carotid artery and the PaO_2_/FiO_2_ (P/F) ratio was calculated to assess systemic oxygenation. In the control group, the P/F ratio significantly declined from baseline to 2 h post-reperfusion, whereas the Ar group exhibited a smaller, non-significant decrease. Data are presented as individual values with the mean ± SEM. * *p* less than 0.05. ns: not significant.

To evaluate systemic oxygenation, arterial blood gas analysis was performed using samples collected from the carotid artery, and the P/F ratio was calculated. In the control group, the P/F ratio significantly declined from 568 ± 12 mmHg pre-ischemia to 272 ± 39 mmHg 2 h post-reperfusion (*p* < 0.05). In contrast, the Ar group showed a smaller, non-significant decrease from 562 ± 17 to 430 ± 48 mmHg (Figure 2).

**Figure 3:**
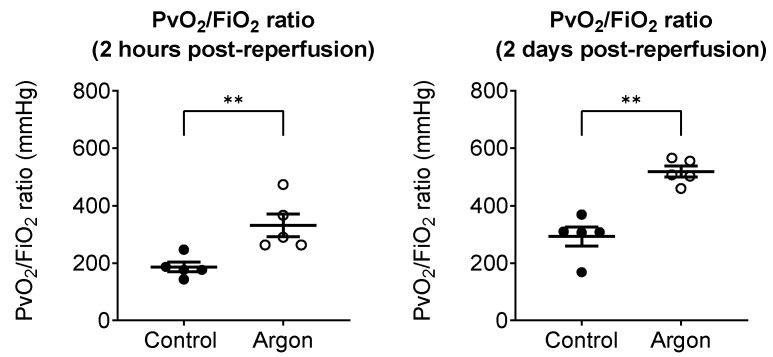
Pulmonary venous oxygenation of the reperfused left lung was assessed using the PvO_2_/FiO_2_ (Pv/F) ratio, obtained from blood sampled from the left pulmonary vein. Two hours post-reperfusion, the argon group showed significantly higher Pv/F ratios than the control group (black dots: control; white circles: Ar group). Two days post-reperfusion, the argon group maintained superior oxygenation with near-complete recovery, whereas the control group exhibited persistently impaired values. Data are presented as individual values with the mean ± SEM. ** *p* less than 0.01.

To evaluate the oxygenation of the reperfused left lung, we used the pulmonary venous oxygenation index (Pv/F ratio), as described in the Methods Section. Two hours post-reperfusion, the Pv/F ratio was significantly higher in the Ar group (331 ± 40 mmHg) than in the control group (186 ± 17 mmHg; *p* < 0.01). A similar trend was observed 2 days post-reperfusion, with Pv/F ratios of 519 ± 19 mmHg in the Ar group and 292 ± 33 mmHg in the control group (*p* < 0.01), indicating near-complete recovery of oxygenation in the Ar group (Figure 3).

### 3.2. Argon Inhalation Reduces Pulmonary Infiltrates After IRI

**Figure 4:**
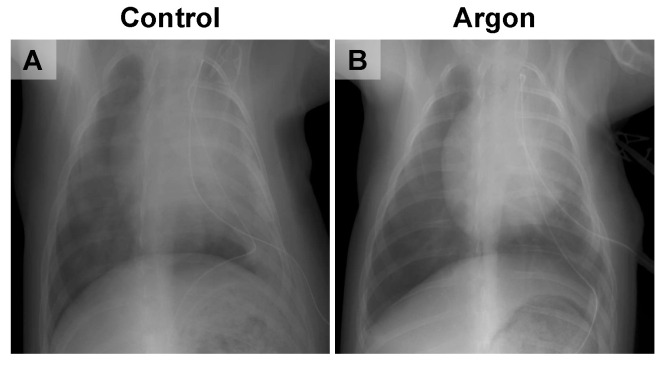
Representative chest X-ray images from the control (**A**) and argon (Ar) groups (**B**). The control group showed marked pulmonary infiltration and reduced lung expansion, whereas the Ar group exhibited preserved lung expansion with only minimal infiltration.

Chest X-rays performed two days post-reperfusion revealed marked pulmonary infiltrates and reduced lung expansion in the control group. In contrast, the Ar group exhibited preserved lung expansion and only minimal infiltration (Figure 4). By day 7, both groups showed signs of recovery and no notable differences were observed. Findings on days 14 and 28 also demonstrated complete radiographic recovery in both groups.

### 3.3. Argon Inhalation Reduces Histopathological Lung Injury

**Figure 5:**
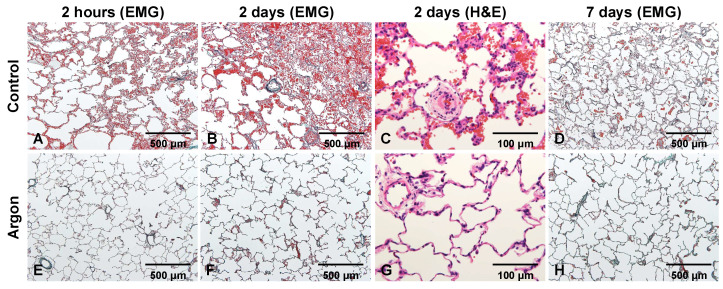
Representative lung sections from the control and argon (Ar) groups 2 h, 2 days, and 7 days post-reperfusion. In the control group (**A**–**D**), EMG and H&E staining showed early neutrophil infiltration, edema, hemorrhage, and fibrin deposition, which became more pronounced on day 2. By day 7, the residual fibrin and alveolar wall injuries persisted. In contrast, the Ar group (**E**–**H**) displayed nearly preserved architecture at 2 h, with only mild edema and limited cellular infiltration on day 2, and substantial resolution of abnormalities by day 7. Hematoxylin and eosin (H&E) and Elastica Masson–Goldner (EMG).

Histopathological analysis of lung biopsy specimens obtained 2 h post-reperfusion in the control group revealed neutrophil infiltration, alveolar edema, hemorrhage, and fibrin deposition (Figure 5A). These changes worsened by day 2, with pronounced edema, hemorrhage, and neutrophil infiltration in alveolar capillaries and pulmonary arterioles (Figure 5B,C). By day 7, fibrin deposition persisted because of alveolar wall damage (Figure 5D). In contrast, the Ar group showed nearly normal histology at 2 h (Figure 5E), with only mild edema and limited cellular infiltration on day 2 (Figure 5F,G). By day 7, most abnormalities had resolved, with minimal residual fibrin (Figure 5H). Findings on days 14 and 28 likewise showed near-complete histological recovery in both groups; therefore, images from these time points are not presented. The histological scores of the lung biopsies are summarized in Table 1.

### 3.4. Argon Inhalation Suppresses Apoptosis in Ischemic Lung Tissue

**Figure 6:**
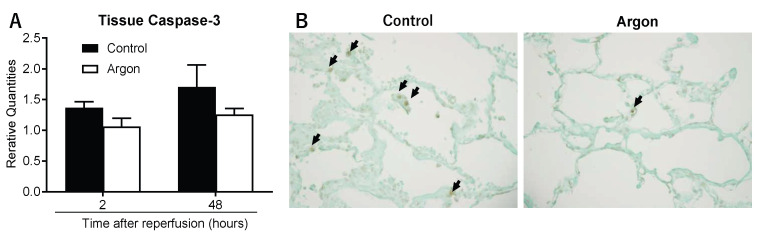
(**A**) Caspase-3 mRNA expression 2 h and 2 days post-reperfusion. Both groups showed increased expression, but the increase was less pronounced in the argon (Ar) group. (**B**) Representative TUNEL staining 2 days post-reperfusion. The number of apoptotic cells (arrows) per high-power field (400×) was markedly lower in the Ar group than in the control group (1.7 ± 0.3 vs. 6.6 ± 0.6 cells per field, respectively; *p* < 0.01).

Caspase-3 mRNA expression was measured to examine apoptosis in tissues. In the control group, expression increased to 1.37 ± 0.10 at 2 h and 1.70 ± 0.36 two days post-reperfusion. Although increases were observed in the Ar group, they were less pronounced (Figure 6A). TUNEL staining at 2 days post-reperfusion indicated a significant reduction in TUNEL-positive cells in the Ar group (Figure 6B). To confirm this finding, we quantified the TUNEL-positive nuclei in the lung tissue 2 days post-reperfusion. The number of apoptotic cells per high-power field (400×) was markedly lower in the Ar group than in the control group (1.7 ± 0.3 vs. 6.6 ± 0.6 cells per field, respectively; *p* < 0.01), providing quantitative support for the anti-apoptotic effect of Ar in this model.

### 3.5. Argon Inhalation Enhances Antioxidant Response

**Figure 7:**
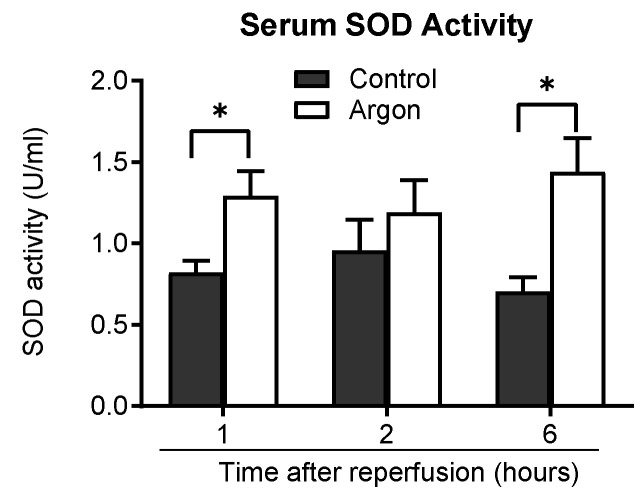
Serum superoxide dismutase (SOD) activity was measured 1, 2, and 6 h post-reperfusion in the control and argon (Ar) groups. The Ar group exhibited consistently higher SOD activity than the control group at all time points, with statistically significant increases at z1 and 6 h post-reperfusion (* *p* less than 0.05). Data are presented as the mean ± SEM.

Serum superoxide dismutase (SOD) activity was measured to assess the antioxidant effects. The Ar group consistently exhibited higher SOD activity than the control group 1, 2, and 6 h post-reperfusion, with significant differences at 1 and 6 h (*p* < 0.05), supporting the antioxidant role of Ar (Figure 7).

### 3.6. Argon Inhalation Shows Limited Anti-Inflammatory Effects

**Figure 8:**
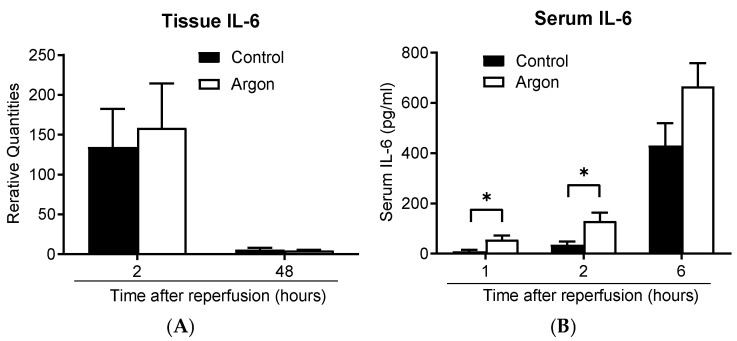
IL-6 mRNA expression in the lung tissue increased 2 h post-reperfusion and declined by 48 h in both groups (**A**). The serum IL-6 concentration increased over time in both groups (**B**). IL-6 levels were significantly lower in the control group than in the argon group 1 and 2 h post-reperfusion (* *p* less than 0.05). Data are presented as the mean ± SEM.

IL-6 mRNA expression in lung tissue increased in both groups 2 h post-reperfusion and decreased by day 2 (Figure 8A). Serum IL-6 concentrations also increased over time in both groups (Figure 8B). Interestingly, IL-6 levels were significantly lower in the control group than in the Ar group 1 and 2 h post-reperfusion. These findings suggest that Ar inhalation did not exert an anti-inflammatory effect under these conditions.

### 3.7. Argon Inhalation Causes No Detectable Adverse Effects

To evaluate the potential systemic toxicity of prolonged Ar inhalation (360 min total), the serum Cre and ALT levels were monitored. No significant differences were found between the two groups throughout the study period, and no adverse effects were observed (Table 2).

## 4. Discussion

In this clinically relevant swine model of warm lung IRI, perioperative inhalation of a 70% Ar and 30% O_2_ mixture markedly attenuated acute lung injury and preserved pulmonary function. Ar-treated animals exhibited improved systemic oxygenation, significantly higher Pv/F ratios reflecting regional gas exchange, and reduced radiographic and histological injuries. These protective effects were supported by decreased apoptosis and enhanced antioxidant responses. Collectively, these findings suggest that Ar exerts potent cytoprotective actions during the reperfusion phase, which represents a major unmet target in current lung transplantation management.

Warm ischemia rapidly depletes ATP, leading to hypoxanthine accumulation. Upon reperfusion, xanthine oxidase converts hypoxanthine into superoxide, causing a substantial oxidative burst and disruption of the alveolar–capillary barrier [8]. This process drives PGD and contributes to CLAD [9]. Pharmacological approaches to mitigate IRI remain limited.

Medical gases have gained attention because inhalation enables targeted delivery to the alveolar–capillary interface with minimal systemic toxicity [24]. Noble gases, previously regarded as inert, have demonstrated organ-protective effects, including anti-apoptotic and antioxidant actions [25]. Argon has shown protective efficacy across multiple organs, including improved outcomes in cerebral IRI [26,27,28], attenuated myocardial infarction with intermittent 70% Ar [14], reduced renal IRI with Ar-saturated perfusate [16], and decreased neuronal injury in large-animal cerebral ischemia models [27]. Porcine kidney perfusion studies have demonstrated protective effects [20].

Previous experimental findings in the lung have predominantly reported a lack of benefit. Martens et al. found no protective effect of Ar in porcine EVLP despite high Ar exposure [21,29]. This discrepancy likely reflects fundamental differences between EVLP and in vivo conditions, particularly regarding oxygen concentration, reperfusion timing, and the presence of intact vascular and inflammatory responses. Our results suggest that the protective effects of Ar become apparent only when these physiological components are preserved, as in the in vivo setting.

Multiple strategies exist for mitigating IRI, including donor selection [30], preservation solutions [31], and pharmacological interventions [32]. EVLP is particularly effective for reconditioning marginal donor lungs [33,34,35]; reducing cytokines such as TNF-α, IL-6, and IL-8; and decreasing DAMP release. However, EVLP acts exclusively during the pre-transplant phase and does not address the reperfusion injury occurring in the recipient. In contrast, Ar can be administered during EVLP and the reperfusion period, precisely when oxidative burst, mitochondrial dysfunction, and apoptosis peak, making it a complementary perioperative adjunct rather than a replacement for EVLP.

The known cytoprotective actions of Ar include both anti-apoptotic and antioxidant mechanisms, and our findings align well with these previously described pathways. Ar has been shown to suppress caspase-3 activation [27], reduce TLR2/4 expression [36], and activate ERK1/2 [28]. Additional studies have demonstrated M2 macrophage polarization [37], SAPK/JNK inhibition and HMGB1 release [15,38], and downregulation of IL-8, which attenuates early neutrophil recruitment [39,40]. Ar may further enhance antioxidant defenses through PI3K–ERK1/2–mTOR signaling and Nrf2-dependent gene induction, including NQO1 and SOD-1 [41]. In this context, the consistently higher SOD levels observed in the Ar group provide experimental support for enhanced antioxidative activity during early reperfusion. As epithelial and endothelial apoptosis drive early lung IRI, the modulation of these pathways offers a mechanistic explanation for the preserved alveolar–capillary integrity and improved Pv/F ratios observed in our study.

In contrast to its anti-apoptotic and antioxidant actions, Ar inhalation did not reduce IL-6 levels; early systemic IL-6 levels were transiently higher in the Ar group. This suggests that the protective effects of Ar in this model predominantly involve antioxidant and anti-apoptotic mechanisms rather than the broad suppression of inflammatory cytokines. The transient rise in IL-6 is consistent with reports that Ar can evoke mild TLR2/4-dependent signaling despite an overall cytoprotective profile [39]. Thus, early cytokine elevation may reflect context-dependent stress signaling rather than harmful inflammation.

Although early systemic IL-6 levels were transiently higher in the Ar group, lung tissue IL-6 mRNA expression did not differ between groups, suggesting that this difference reflects a systemic rather than a lung-restricted response. Importantly, Argon’s protective effects—improved oxygenation, reduced apoptosis, and enhanced antioxidant activity—were preserved despite this cytokine pattern, indicating that its benefits in warm lung IRI are mediated predominantly through anti-apoptotic and antioxidant pathways. Prior reports describing the anti-inflammatory effects of argon were conducted in retinal [36] or neuronal models [40] with localized and barrier-restricted inflammation, whereas the present large-animal lung IRI model evokes a broader systemic inflammatory activation. These organ- and model-dependent differences likely explain the distinct IL-6 kinetics observed in our study.

Argon’s protective effects have been reported to be dose- and time-dependent. In a cardiac arrest model, 70% Ar provided greater neuroprotection than 40% [42]. Similarly, 25–75% Ar showed time- and dose-dependent retinal protection [43], and proper timing and duration were crucial for neuroprotection in a transient middle cerebral artery occlusion mouse model [44]. These findings suggest that optimizing the Ar concentration and exposure remains important for translation.

In this study, we selected 70% Ar because it represents the highest concentration that can be safely administered while maintaining an FiO_2_ of 0.30. The 360-min inhalation period was chosen to cover the entire peri-reperfusion window and was based on our previous large-animal experiments in which 360 min of carbon monoxide (CO) inhalation effectively attenuated lung IRI [22]. Because the oxidative burst that initiates early reperfusion injury peaks within the first minutes to hours after reperfusion [8,9], continuous exposure throughout this window is considered important. In the present study, antioxidant and anti-apoptotic effects were evident at both 2 h and 2 days after reperfusion, supporting the adequacy of this exposure duration for suppressing ROS-mediated injury. Nevertheless, the optimal combination of Ar concentration and treatment duration remains unknown, and future studies will explore alternative dosing regimens.

A major strength of this study is the use of a clinically relevant large-animal model with a 28-day follow-up, whereas many prior Ar studies assessed only the early time points. Normoxic administration enhances its feasibility. The chemical inertness of Ar provides advantages over gases such as nitric oxide, CO, hydrogen, and hydrogen sulfide, which require strict safety management [45,46,47,48,49]. Ar is non-toxic, non-flammable, and simple to administer, making it suitable for perioperative use and potentially compatible with EVLP circuits. Synergistic combinations with other medical gases have also been explored [50].

This study has some limitations. Cold preservation has not been evaluated [51], although standard graft preservation relies on cold storage with dextran-based solutions and oxygenated cooling [52,53]. Warm and cold IRI differ markedly in inflammatory patterns and metabolic suppression [54,55]; thus, the effects of Ar under cold storage conditions warrant further investigation. Moreover, although early (2 h) and later (48 h) apoptotic changes were assessed, intermediate time points might also provide additional detail regarding the temporal progression of apoptosis. Other limitations include the modest sample size, the assessment of only one Ar concentration and exposure duration, and the absence of transcriptomic or long-term CLAD-like analyses.

## 5. Conclusions

Perioperative Ar inhalation significantly attenuated warm lung IRI in miniature swine by reducing apoptosis, enhancing antioxidant responses, and preserving pulmonary oxygenation. By targeting the reperfusion phase, which is unaddressed by donor-side strategies such as EVLP, Ar may represent a practical and safe adjunct for lung transplantation. Further mechanistic and translational studies, including validation in large-animal transplantation models, are warranted to determine optimal dosing and timing, which will allow for integration into clinical practice. Such efforts may facilitate the development of strategies to improve graft quality and ultimately expand the pool of transplantable organs.

## Figures and Tables

**Table 1:** Histological scores of lung biopsies based on light microscopy.

	2 h Post-Reperfusion		2 Days Post-Reperfusion	
	Control	Argon	Control	Argon
Infiltrating cells	1.3 ± 0.1	0.7 ± 0.1 *	1.6 ± 0.2	0.8 ± 0.2 *
Intra-alveolar edema	0.7 ± 0.1	0.2 ± 0.1 *	1.5 ± 0.2	0.7 ± 0.1
Fibrin exudation	0.6 ± 0.2	0.0 ± 0.0 *	2.1 ± 0.3	0.8 ± 0.1 **
Intra-alveolar hemorrhage	1.2 ± 0.3	0.5 ± 0.2	1.7 ± 0.3	0.8 ± 0.1

Data are shown as mean ± SEM. * *p* less than 0.05. ** *p* less than 0.01.

**Table 2:** Changes in kidney and liver function.

**Cre (mg/dL)**					
	Baseline	Day 2	Day 7	Day 14	Day 28
Control	0.8 ± 0.1	0.9 ± 0.0	0.6 ± 0.1	0.7 ± 0.0	0.8 ± 0.0
Argon	0.9 ± 0.1	0.9 ± 0.0	0.7 ± 0.0	0.7 ± 0.0	0.9 ± 0.0
**ALT (U/L)**					
	Baseline	Day 2	Day 7	Day 14	Day 28
Control	24 ± 1	47 ± 4	34 ± 2	28 ± 1	24 ± 1
Argon	26 ± 2	57 ± 8	47 ± 6	31 ± 2	24 ± 1

Cre: creatinine, ALT: alanine aminotransferase.

## Data Availability

The original contributions presented in this study are included in this article. Further inquiries should be directed to the corresponding author.

## Abbreviations

The following abbreviations are used in this manuscript:

Ar	Argon
DCD	Donation after circulatory death
IRI	Ischemia–reperfusion injury
PGD	Primary graft dysfunction
CLAD	Chronic lung allograft dysfunction
H&E	Hematoxylin and eosin
EMG	Elastica Masson–Goldner
TUNEL	Terminal deoxynucleotidyl transferase-mediated UTP-biotin nick end labeling
Cre	Creatinine
ALT	Alanine aminotransferase
SOD	Superoxide dismutase
cDNA	Complementary DNA
RPL4	Ribosomal protein L4
SEM	Standard error of the mean
P/F ratio	PaO_2_/FiO_2_ ratio
Pv/F ratio	PvO_2_/FiO_2_ ratio
EVLP	Ex vivo lung perfusion
HMGB1	High-mobility group box-1
CO	Carbon monoxide
